# DSDBASE 2.0: updated version of DiSulphide dataBASE, a database on disulphide bonds in proteins

**DOI:** 10.1093/database/baac005

**Published:** 2022-03-01

**Authors:** Neha V Kalmankar, Murugavel Pavalam, Sowmya Indrakumar, Narayanaswamy Srinivasan, Ramanathan Sowdhamini

**Affiliations:** National Centre for Biological Sciences, Tata Institute of Fundamental Research (TIFR), GKVK Campus, Bellary Road, Bengaluru, Karnataka 560065, India; The University of Trans-Disciplinary Health Sciences and Technology (TDU), #74/2, Jarakabande Kaval, Post Attur, Via Yelahanka, Bengaluru, Karnataka 560064, India; National Centre for Biological Sciences, Tata Institute of Fundamental Research (TIFR), GKVK Campus, Bellary Road, Bengaluru, Karnataka 560065, India; Molecular Biophysics Unit, Indian Institute of Science, Bengaluru, Karnataka 560012, India; Molecular Biophysics Unit, Indian Institute of Science, Bengaluru, Karnataka 560012, India; National Centre for Biological Sciences, Tata Institute of Fundamental Research (TIFR), GKVK Campus, Bellary Road, Bengaluru, Karnataka 560065, India; Molecular Biophysics Unit, Indian Institute of Science, Bengaluru, Karnataka 560012, India; Institute of Bioinformatics and Applied Biotechnology, Biotech Park, GN Ramachandran Road, Electronics City Phase 1, Bengaluru, Karnataka 560100, India

## Abstract

Disulphide bonds are stabilizing crosslinks in proteins and serve to enhance their thermal stability. In proteins that are small and rich in disulphide bonds, they could be the major determining factor for the choice of conformational state since their constraints on appropriate backbone conformation can be substantial. Such crosslinks and their positional conservation could itself enable protein family and functional association. Despite the importance of the field, there is no comprehensive database on disulphide crosslinks that is available to the public. Herein we provide information on disulphides in DSDBASE2.0, an updated and significantly expanded database that is freely available, fully annotated and manually curated database on native and modelled disulphides. The web interface also provides several useful computational tools that have been specifically developed for proteins containing disulphide crosslinks. The modelling of disulphide crosslinks is performed using stereochemical criteria, coded within our Modelling of Disulphides in Proteins (MODIP) algorithm. The inclusion of modelled disulphides potentially enhances the loop database substantially, thereby permitting the recognition of compatible polypeptide segments that could serve as templates for immediate modelling. The DSDBASE2.0 database has been updated to include 153,944 PDB entries, 216,096 native and 20,153,850 modelled disulphide bond segments from PDB January 2021 release. The current database also provides a resource to user-friendly search for multiple disulphide bond containing loops, along with annotation of their function using GO and subcellular localization of the query. Furthermore, it is possible to obtain the three-dimensional models of disulphide-rich small proteins using an independent algorithm, RANMOD, that generates and examines random, but allowed backbone conformations of the polypeptide. DSDBASE2.0 still remains the largest open-access repository that organizes all disulphide bonds of proteins on a single platform. The database can be accessed from http://caps.ncbs.res.in/dsdbase2.

## Overview

Disulphide (S–S) bonds, i.e. the covalent crosslinks between thiol groups of two cysteine residues, are recognized means of stabilizing native and folded proteins (1). Such disulphide crosslinks are known to entropically destabilize the unfolded states of a polypeptide, limit the mobility and thereby increase the stability of the folded form and the native state of a polypeptide (2, 3). Approximately 20% of human proteins contain disulphide bonds (4). Proteins with such domains are often referred to as disulphide-rich domains, and they can exist both as single-domain structures and as substructures within larger polypeptides. Such domains are typically present in extracellular proteins and peptides that include growth factors, hormones, enzymes, etc. A vast majority of disulphide bonds are also present in exterior cell surface proteins, such as immune-cell receptor CD4, integrin receptor α_IIb_β_3_, HIV-1 envelope glycoprotein gp120, and several others. An important exception is glutathione, which forms a dimer via a disulphide bond in the interior of the cell in order to regulate and maintain cellular redox environment together with other redox-active compounds and also to inactivate reactive oxygen species within the cell. Several small, disulphide-rich systems are also popularly known as bioactive peptides ranging from toxins, cyclotides, insulin and related growth factors, trypsin inhibitors to vasoconstrictors, antibacterial peptides, oxytocin, etc. Despite their abundance in sequence databases and biomedical importance, the three-dimensional structures are not available for many such proteins and peptides. Structural information can be derived by finding homologous entries in protein structure databanks based on conserved cysteine connectivity patterns. However, using existing methods such as PSI-BLAST (5) generally tend to be unreliable for small peptides due to high sequence identity to a wide variety of structural hits, thereby increasing the rate of false positives. Here, we introduce an updated database—DSDBASE2.0—that provides information on native disulphides, along with modelled disulphides, and those which are stereochemically possible between pairs of residues in a protein. We had previously developed the database for this purpose—DSDBASE (6), which has been widely used and remains as one of the few available resources for such purposes. The current database has not only been substantially enhanced to include more recent PDB entries (Tables 1 and 2), it also provides a resource for user-friendly search of multiple disulphide bond-containing loops, along with their annotation of function using GO and active-site residue information. Additionally, using RANMOD it is now possible to obtain three-dimensional models of disulphide-rich peptides. The RANMOD (7) tool generates and examines random, but allowed backbone conformations that a polypeptide can assume.

**Table 1.** Distribution of disulphide bonds in DSDBASE version 2.0

Dataset^a^	No. of proteins	Native disulphides	Modelled disulphides	Total disulphides
Non-redundant_30 or nr-db-30% (Jan 2021)	26 638	20 932	2 282 752	2 303 684
Non-redundant_90 or nr-db-90% (Jan 2021)	43 200	51 932	5 012 608	5 064 540
Full database or fulldb (Jan 2021)	153 944	216 096	20 153 850	20 369 946

a nr-db-30%, nr-db-90% and fulldb represent databases of native and modelled disulphide bonds derived from non-redundant set of proteins with 30% and 90% sequence identity cut-offs and full PDB release, respectively, derived from PDB (January 2021).

**Table 2.** Distribution of disulphide bonds in the DSDBASE previous version

Dataset^a^	No. of proteins	Native disulphides	Modelled disulphides	Total disulphides
Non-redundant_25 or nr-db-25% (April 2001)	1131	950	46 568	47 518
Full Database or fulldb (PDB April 2001)	13 513	22 588	1 491 597	1 514 185
Non-redundant_25 or nr-db-25% (April 2002)	1758	1413	70 887	72 300
Full database or fulldb (PDB July 2002)	17 163	28 141	1 973 898	2 002 039
Non-redundant_25 or nr-db-25% (April 2003)	2849	2170	147 722	149 892
Full database or fulldb (PDB April 2003)	19 612	31 657	2 353 960	2 385 617

a nr-db-25% and fulldb represent databases of native and modelled disulphide bonds derived from non-redundant set of proteins with 25% sequence identity cut-off and full PDB release, respectively, derived from different PDB datasets (April 2001, April 2002, July 2002 and April 2003 releases).

In theory, the stability of proteins can be reduced by the removal of native disulphide bonds. On the other hand, structural stability or rigidity of proteins can be improved by engineering new disulphide bonds. Several strategies, both experimental and computational, and empirical design rules have been proposed to introduce non-native disulphide bonds into protein structures in an attempt to improve their properties. However, successfully engineering a disulphide bond to increase stability still remains a significant challenge. Computationally, tools such as Disulfide by Design (8), SSBOND (9), MODIP (10), and limited others have been developed for the identification of ‘hot-spot’ sites suitable for strainless disulphide bond introduction (11, 12). In the updated database, the modelling of disulphides is performed by the MODIP algorithm, which essentially identifies pairs of residues where stereochemically covalent crosslinks can be accommodated without any strain. These crosslinks are further graded (A, B or C) according to their stereochemical quality. Moreover, it is observed that it is more effective to search in the current database for suitable templates and structural homologues to model disulphide-rich systems. The disulphide bond connectivity information is supplied by the user and the tool searches for substructures in the database to propose 3D models of disulphide-rich polypeptides, in turn, allowing the design of site-directed mutants for enhanced stability of the protein. This forms another useful application of the database.

## Structure of the updated database

**Figure 1.**
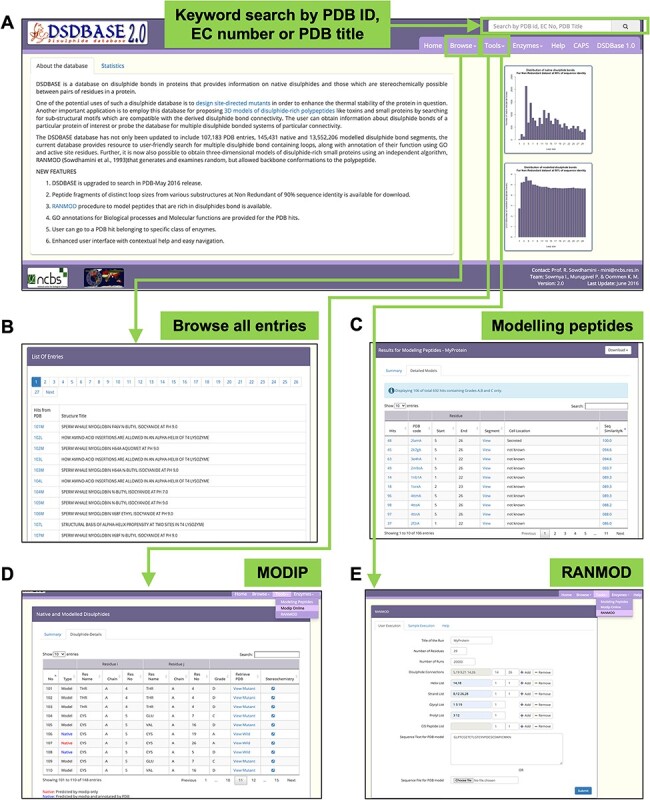
DSDBASE 2.0 database organization and features. Different features of the database have been shown here. (A) Database browser. The users can browse through the different modules of DSDBASE. The user can also perform keyword search by PDB ID, EC number or PDB title. The distribution of native and modelled disulphide bonds based on loop sizes is provided. (B) List of all entries. The user can also download peptide fragments of distinct loop sizes from various substructures from nr-db-90% (90% sequence identity). (C) Modelling peptide feature. (D) RANMOD feature.

DSDBASE2.0 can be accessed from http://caps.ncbs.res.in/dsdbase2. The present release considers all entries in PDB-Jan 2021 release. The current database can be employed to search for loops with multiple disulphide bonds, along with annotation of their biological processes and molecular function, and subcellular localization of the query. A keyword search, PDB code search and EC number search options are made available. All possible pairs of residues that can strainlessly accommodate a disulphide bond and the stereochemical parameters of the disulphide bond are listed. Furthermore, there is a possibility to download all sub-structural motifs having specific loop sizes as well. Modelling peptides, an online search program to identify the nearest sub-structural homologue is provided. The input for that is the disulphide bond connectivity information for probing the database—and all sub-structural motifs with similar disulphide bonding pattern that can accommodate the restraints will be recognized. The modelling of disulphides is performed using MODIP algorithm. For the missing PDB entries, modelling can be performed by providing a PDB file as the input. Figure 1 shows various screenshots from the database.

## Database statistics

The DSDBASE2.0 database has been updated to include 153 944 PDB entries, 216 096 native and 20 153 850 modelled disulphide bond segments (Table 1). DSDBASE2.0 has also been updated to record 20 369 946 protein substructures that have stereochemical compatibility to accommodate disulphide bonds (Tables 3 and 4).

**Table 3.** Number of substructures recorded in DSDBASE version 2.0

	Full database	Non‐redundant_30 (nr_30) database	Native of nr_30 database
No. of proteins	153 944	26 638	4544
No. of SS bonds	20 369 946	2 303 684	20 932
Grade A	2 625 229 (13%)	291 084 (13%)	12 702 (61%)
Grade B	5 431 859 (26%)	609 450 (26%)	3230 (15%)
Grade C	6 220 158 (31%)	713 542 (31%)	3027 (14%)
Grade D ^a^	6 092 700 (30%)	689 608 (30%)	1973 (10%)

a Cα–Cα and Cβ–Cβ distances were compatible but sulphur could not be fixed geometrically.

**Table 4.** Number of substructures recorded in DSDBASE previous version

	Full database	Non‐redundant_30 (nr_30) database	Native of nr_30 database
No. of proteins	19 612	2849	766
No. of SS bonds	2 385 617	149 892	2170
Grade A	307 003 (12%)	19 591 (13%)	1321 (61%)
Grade B	633 431 (26%)	39 804 (27%)	341 (16%)
Grade C	728 374 (30%)	90 497 (60%)	508 (23%)
Grade D^a^	716 809 (29%)	–	–

a Cα–Cα and Cβ–Cβ distances were compatible but sulphur could not be fixed geometrically.

## Features and interfaced tools

### MODIP online—designing site-directed mutants

**Figure 2.**
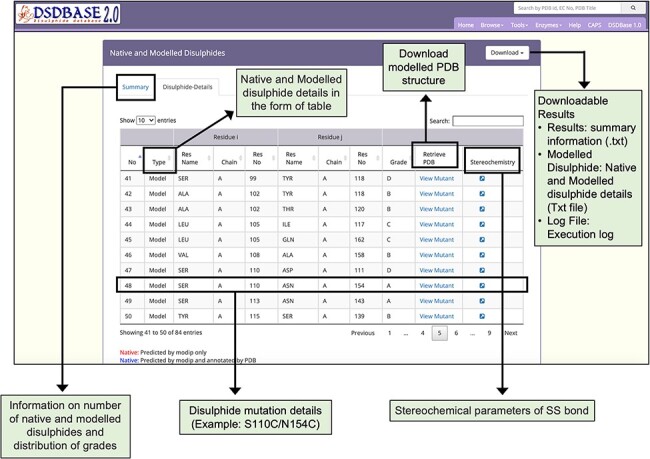
Results page for modelling of disulphide bonds in proteins (MODIP). A snapshot of the search output page shows the summary of native and modelled disulphides along with the grades and details of native and modelled disulphide in the form of table. The user should provide a structure (PDB format) for running MODIP procedure.

MODIP is available online and can be used to examine sites where disulphides can be introduced strainlessly (10). Figure 2 depicts the output page of the MODIP run for a test protein for which stability data for disulphide engineered mutants were available. After the input of N, Cα and Cβ coordinates from the protein crystal structure, the programme evaluates all possible Cα-Cα distances and the corresponding Cβ-Cβ distances. Those pairs of residues whose Cα-Cα distance is ≤6.5 Å and Cβ-Cβ distance is ≤4.5 Å are selected for fixation of a sulphur atom at the two Cβ atom positions. Furthermore, the program calculates the S–S bridge parameters. A gradation scheme is used to evaluate the modelled disulphide, rank them based on certain stereochemical parameters such as dihedral angles and S–S bond distance (10). Thus, a disulphide bridge modelled with dihedral angles and S-S bond distance within the accepted range is graded as ‘A’. Grade ‘B’ are those disulphide bridges where it is geometrically suitable to form the S–S covalent bond but with somewhat distorted stereochemistry. Sites that are spatially too close to form a disulphide bridge is graded as ‘C’.

### RANMOD—random conformation to polypeptide backbone

**Figure 3.**
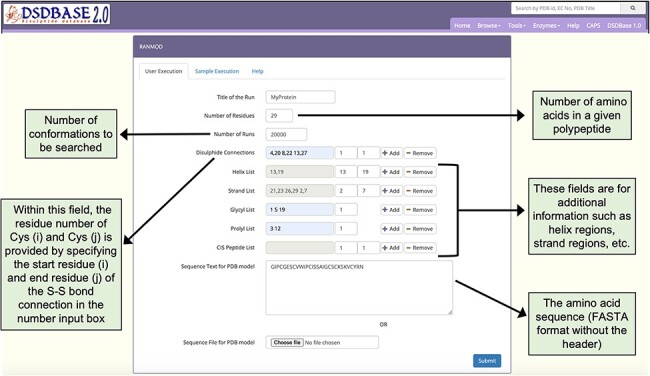
Snapshot of the input page for RANMOD (Random Conformation to Polypeptide Backbone) program. The user should provide a sequence (FASTA format) and disulphide connections information for running RANMOD procedure.

The independent algorithm, RANMOD, has been added as a new feature (7). Figure 3 depicts the output page of the RANMOD run for a test protein. This method assigns a large number of permitted backbone conformations in a random fashion to the polypeptide and identifies models that could accommodate strainless disulphide bridges. It uses the MODIP program to model the disulphide bridges (10). This method is beneficial for modelling disulphide-rich peptides for which there is no structure reported. This procedure has been applied on two and three disulphide bond-containing peptides and found to give rise to several stereochemically acceptable structures. The search is time efficient; for instance, it takes less than 30 seconds to examine 200 000 conformations before finally converging at a stereochemically acceptable structure. Depending on the input seed number, RANMOD assigns random values to each of the backbone torsion angles at each residue along the polypeptide chain. This implies various possible conformations with distinct seed number. The present version can distinguish glycine, proline and cysteine residues, and the rest of the residues are treated as alanine residues. It is up to the user to identify the ‘best’ model as the present version does not rank the proposed models.

### Modelling polypeptides

**Figure 4.**
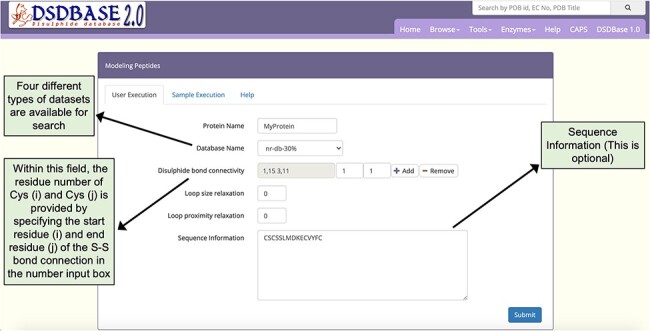
Snapshot of the input page for Modelling peptides. The user can probe the database for multiple disulphide-bonded systems of particular connectivity and get possible conformations for that segment with the option to provide partial or no sequence information.

A search engine for modelling disulphide-rich peptides has been added as a feature (13). Users can probe the database for multiple disulphide bonded systems of particular connectivity and get possible conformations for that segment with the option to provide partial or no sequence information. Figure 4 depicts the output page of the search engine. This tool searches against a non-redundant database for homologues based on disulphide connectivity and/or sequence similarity details input by the user. The searches can be performed against the full PDB database (fulldb) or the non-redundant PDB database (nr-db-30%; 30% sequence identity and nr-db-90%; 90% sequence identity) or a subset of native disulphides of the non-redundant set of proteins (nr-native-30%). The output generates protein or peptides hits or sub-structural motifs of a protein that shares a similar connectivity pattern as that of the query. The predicted subcellular location of the query is also provided, and the native disulphide bonds and redox-active disulphide bonds are highlighted. The option to download the coordinate files of the hits and a graphical view of the segment via JSmol are also made available.

## Scope of the database/case studies

### MODIP

#### Xylanase

**Figure 5.**
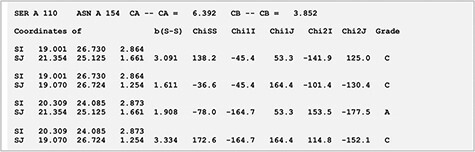
Stereochemistry of the predicted mutation site (S110C-N154C) for disulphide bridge modelling in the case of xylanase (PDB ID: 1XYP).

Xylanases are the most important xylan-degrading enzymes that are responsible for the hydrolysis of β-1,4 bonds in plant xylan, the main component of the hemicellulose. MODIP program was used to predict sites in an endo-β-1,4-xylanase from *Trichodrema reesei* (PDB ID: 1XYP) where cysteine mutagenesis can be sterically introduced, thereby imparting stability to the protein (14). The analyses revealed a key site of mutagenesis at positions S110C-N154C (Figure 2). The introduction of a disulphide bond at these sites is known to increase the thermodynamic stability of the protein, and our prediction from the list supports this assertion (15). The disulphide bridge was modelled with dihedral angles within an ideal range of values and was assigned grade ‘A’ based on the stereochemical parameters such as dihedral angles and S-S bond distance (Figure 5).

### RANMOD

#### Cyclotide

**Figure 6.**
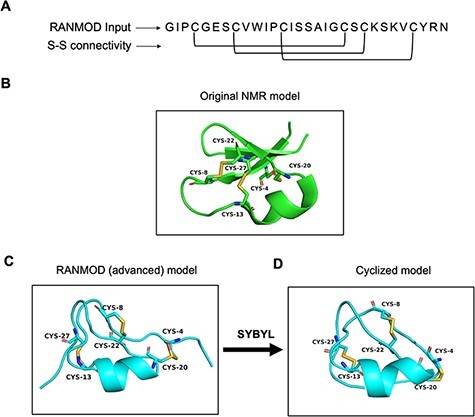
Model generated using RANMOD program for test case of cycloviolacin O2. (A) The input sequence of cycloviolacin O2 from *Violoa odorata* (PDB ID: 2KNM) and disulphide connectivity information. (B) The native NMR model (the first model of the ensemble) with the disulphide connectivity highlighted. (C) Cartoon representation of the generated model using secondary structural clues. (D) Cartoon representation of the cyclized and energy minimized model. Green and cyan cartoon corresponds to conformations of native template and modelled structures, respectively.

Cyclotides are plant-derived, gene-encoded, triple disulphide, macrocyclic peptides of 26–37 residues length (Figure 6A and B). They are characterized by a cyclic cystine-knot motif, making them ultra-stable peptides against thermal, chemical and enzymatic degradation (16, 17). A single model was generated using the RANMOD tool for the cycloviolacin O2 sequence (PDB ID: 2KNM) (18) with 200 000 number of runs and secondary structural clues provided in the input (advanced modelling) (Figure 6C). The grades of the disulphide bridges for the model is as follows: Cys4-Cys20 Grade B, Cys8-Cys22 Grade C and Cys13-Cys27 Grade C. As cyclotide is naturally a cyclic peptide, the N- and C-terminal residues had to be cyclized. This feature is not available within the RANMOD tool; hence, we were able to generate the cyclizable conformation of cyclotide sequence externally using SYBYL software package version 7.1 (Tripos International, St. Louis, USA). The model generated by RANMOD was further energy minimized using Tripos forcefield using the steepest descent method. As evident in Figure 6B and D, the Cα deviation between RANMOD model and the native NMR model of cycloviolacin O2 sequence is bare minimum, and the models showed an overall topological RMSD of ∼6.7 Å.

#### Conotoxin

**Figure 7.**
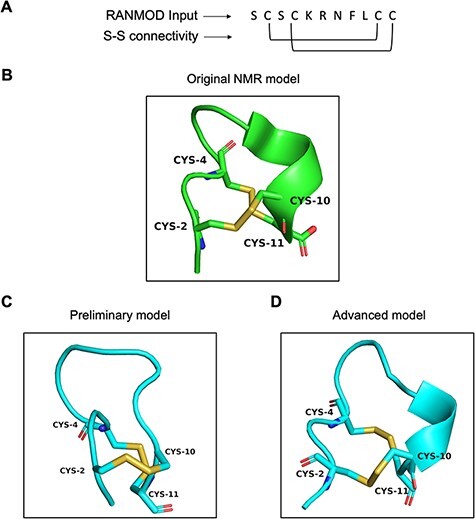
Model generated using RANMOD program for test case of conotoxin pc16a. (A) The input sequence of conotoxin pc16a (PDB ID: 2LER) and disulphide connectivity information. (B) The native NMR model (the first model of the ensemble) with the disulphide connectivity highlighted. (C) Cartoon representation of the generated model using no secondary structural clues (preliminary modelling). (D) Cartoon representation of the generated model using secondary structure information (advanced modelling). Green and cyan cartoon corresponds to conformation of native template and modelled structures, respectively.

Conotoxin is a group of disulphide-rich, neurotoxic peptides present in the venom of marine cone snails. Their size typically ranges from 8–45 amino acid residues and can possess one to five disulphide bonds (19). Here, we demonstrate the structural modelling of a novel conotoxin, pc16a (PDB ID: 2LER), the first peptide characterized from a South African cone snail *Conus pictus* of 11 residues sequence length (Figure 7A) (20). Conotoxin pc16a contains two disulphide bonds (Cys2-Cys10 and Cys4-Cys11), which make the interlocked loops (Figure 7B). In the first attempt of modelling (preliminary), no secondary structural information was provided, except the number of residues, the disulphide bond connectivity information and the full sequence of the peptide. This procedure, which involved 200 000 number of conformations to be searched, generated nine plausible random conformations for conotoxin pc16a (best conformation is highlighted in Figure 7C). In the second attempt (advanced modelling), along with the sequence information, disulphide bond connectivity and peptide length information, any known secondary structural information was also provided prior to the run. This procedure (200 000 runs) generated a single plausible random conformation for conotoxin pc16a (Figure 7D). As the additional input parameters to the modelling procedure increased, structural similarity to the native template structure increased (Figure 7B and D). It should be noted that generating conformations having interlocked disulphide loops is more difficult and complex compared to independent/loop-within-loop disulphide bridges. It was satisfying to note that several local structural features identified in native structures were indeed observed in the conformations obtained by the RANMOD procedure. These findings suggest strong agreement of the overall folding of the conotoxin model to the native structure originally proposed.

#### Buckwheat trypsin inhibitor

**Figure 8.**
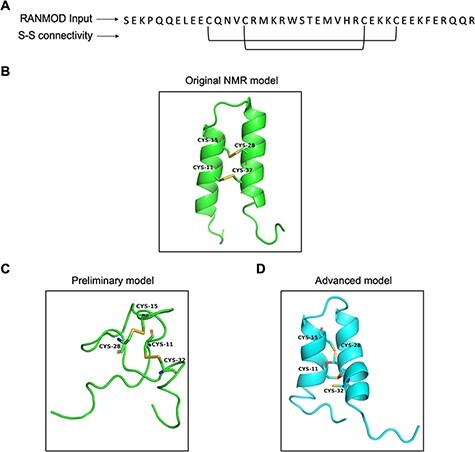
Model generated using RANMOD program for test case of buckwheat trypsin inhibitor BWI-2c. (A) The input sequence of BWI-2c (PDB ID: 2LQX). and disulphide connectivity information. (B) The native NMR model (the first model of the ensemble) with the disulphide connectivity highlighted. (C) Cartoon representation of the generated model using no secondary structural clues (preliminary modelling). (D) Cartoon representation of the generated model using secondary structure information (advanced modelling). Green and cyan cartoon corresponds to conformation of native template and modelled structures, respectively.

Trypsin inhibitors are types of serine protease inhibitors that reduce the biological activity of trypsin. A new peptide trypsin inhibitor (PDB ID: 2LQX) named BWI-2c was obtained from buckwheat (*Fagopyrum esculentum*) seeds by sequential affinity, ion exchange and reversed-phase chromatography (21). The peptide is 41 amino acid residues long and a larger system compared to the previous cases (conotoxin and cyclotide) and the three-dimensional structure was determined by NMR spectroscopy (Figure 8A). It contains four cysteines involved in two intramolecular disulphide bonds at (Cys11-Cys32 and Cys15-Cys28), forming the smaller disulphide loop within the bigger loop (Figure 8B). We performed preliminary modelling without any secondary structural hints and search 200 000 number of conformations. This attempt generated a single model that did not share significant structural homology with the native structure (Figure 8C). It was clear at this stage that providing secondary structural hints is imperative given the long stretches of helices known to be present in the structure of BWI-2c. Hence, we performed advanced modelling by specifying secondary structural clues i.e. helices between residues 5–16 and 21–35. Modelled BWI-2c structure when compared with the native template structure indicates that the modelled structure adopts a similar fold. The secondary structural elements as seen in the native NMR model and as specified prior to the run were indeed observed in the conformation obtained by RANMOD procedure (Figure 8D).

### Modelling disulphide‐rich polypeptides

#### Disulphide connectivity 4–19, 9–21 and 14–26

**Figure 9.**
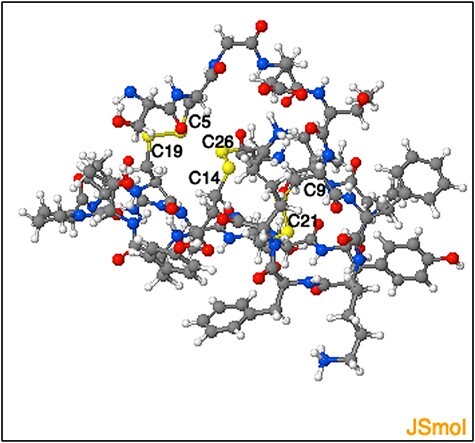
Representation of the three-dimensional structure segment obtained using ‘Modelling peptide’ tool. The three-dimensional structure segment of the homologue identified by querying ‘C4 - C19, C9- C21 and C14 - C26’ disulphide connectivity. The cysteine residues and the corresponding disulphide bridges are highlighted.

The DSDBASE2.0 server can be used for modelling disulphide-rich peptides with a particular disulphide bond connectivity by searching the query segment against the database for structures bearing similar connectivity. We used the ‘Modelling peptides’ tool for searching structural homologues having the disulphide connectivity C4-C19, C9-C21 and C14-C26, and provided the sequence of a cyclotide Cter M (GLPTCGETCTLGTCYVPDCSCSWPICMKN). We then searched against the full PDB database (fulldb). The resulting output displayed a total of 184 hits containing all grades and the top matching hit as PDB ID 2FQA (chain A) with a sequence similarity of 76% with that of the query sequence. The output also provides the subcellular location of the protein hits as extracted from PDB files. The top hit structure we obtained corresponds to a linear cyclotide -Violacin A from *Viola odorata* and contains the exact same disulphide connectivity with that of the query sequence. The ability of the tool to categorically predict a near structural homologue based on the three disulphide-bonded system is noteworthy. Figure 9 shows the segment output feature of the ‘Modelling peptides’ tool. This feature provides the user with the PDB file for the matched segment and can be viewed using JSmol view.

## Discussion and conclusions

To the best of our knowledge, DSDBASE is the first database of its kind that organizes all disulphide bonds of proteins in one platform. A total of 153,944 PDB entries, 216,096 native and 20,153,850 modelled disulphide bond segments from PDB January 2021 release have been included in the updated DSDBASE2.0 database. Furthermore, the current database also provides tools for a user-friendly search of multiple disulphide bond containing loops, along with their functional annotation using GO and subcellular localization of the query. Additionally, an independent algorithm - RANMOD has been added as a useful tool into the webserver. By the very nature of the RANMOD procedure, with constraints on disulphide bond connectivity, multiple conformations are proposed as 3D models of the polypeptide. By the very nature of the RANMOD procedure, with constraints on disulphide bond connectivity, multiple conformations are proposed as 3D models of the polypeptide. In the future, efficient computational methods can be applied to fix side chains of all the residues. Using the installed search engine for modelling disulphide-rich polypeptides, it is also possible to search the non-redundant databases using particular disulphide bond connectivity involving one or more disulphide bonds. The output is a list of proteins or peptides or sub-structural motifs of a protein bearing similar cysteine connectivity patterns. In the future, we wish to update the database further by writing algorithms that can generate models using sequence alignment as an input. Furthermore, within the RANMOD program, we can incorporate computational methods that perform energy minimization routines of the proposed models. This will help the users to rank the proposed models.
